# Crossing length scales: X-ray approaches to studying the structure of biological materials

**DOI:** 10.1107/S2052252524007838

**Published:** 2024-08-28

**Authors:** Tilman A. Grünewald, Marianne Liebi, Henrik Birkedal

**Affiliations:** a Aix Marseille Univ, CNRS, Centrale Med Institut Fresnel Marseille France; b Photon Science Division Paul Scherrer Institute Villigen PSI5232 Switzerland; c Institute of Materials École Polytechnique Fédérale de Lausanne 1015 Lausanne Switzerland; d Department of Chemistry & iNANO Aarhus University Gustav Wieds Vej 14 Aarhus8000 Denmark; UCL, United Kingdom

**Keywords:** X-ray scattering, biomaterials, biomineralization, coherent X-ray diffraction, hierarchical structuring

## Abstract

Biological materials have outstanding properties. With ease, challenging mechanical, optical or electrical properties are realised from comparatively ‘humble’ building blocks. The key strategy to realise these properties is through extensive hierarchical structuring of the material from the millimetre to the nanometre scale in 3D. Though hierarchical structuring in biological materials has long been recognized, the 3D characterization of such structures remains a challenge. To understand the behaviour of materials, multimodal and multi-scale characterization approaches are needed. In this review, we outline current X-ray analysis approaches using the structures of bone and shells as examples. We show how recent advances have aided our understanding of hierarchical structures and their functions, and how these could be exploited for future research directions. We also discuss current roadblocks including radiation damage, data quantity and sample preparation, as well as strategies to address them.

## Abstract 2

Biological materials obtain their properties through hierarchical structuring. Understanding such materials calls for multimodal and multiscale approaches. Based on two example systems, bone and shell, we discuss current analytical approaches, their capabilities and limits, and how to tie them together to fully cover the different length scales involved in understanding materials’ functions. We will further discuss advances in this area and future developments, the possible roadblocks (radiation damage, data quantity, sample preparation) and potential ways to overcome them.

## 1. Introduction

Biological materials play multiple roles in biology, have immense impact on the surface chemistry of Earth (*e.g.* through CO_2_ fixation by CaCO_3_-forming organisms), are essential for human health and serve as inspiration for the design of bioinspired materials. Though they display immense variability in chemical design, a widespread feature is their hierarchical structure (Chen *et al.*, 2020 ▸; Eder *et al.*, 2018 ▸; Bechthold & Weaver, 2017 ▸; Reznikov *et al.*, 2014 ▸, 2016 ▸, 2018 ▸; Barthelat *et al.*, 2016 ▸; Wegst *et al.*, 2015 ▸; Meyers *et al.*, 2013 ▸; Fratzl & Barth, 2009 ▸; Rodriguez–Palomo *et al.*, 2023 ▸; Wittig & Birkedal, 2022 ▸; Buss *et al.*, 2022 ▸; Weiner & Wagner, 1998 ▸). Indeed, structuring across several length scales defines mechanical performance. For example, this can afford actuation in plants by cell wall swelling that generates actuation due to specifically oriented cellulose microfibril motifs, allowing branches to curve upwards or pine cones to change shape (Fratzl & Barth, 2009 ▸). The hierarchical – and often composite – structure of such materials make them very challenging to study. In particular, mineralized composites, where an organic phase templates biomineralization, are of high interest and require techniques that can bridge the crystallographic ångström length scale to the macroscopic length scale of biological structures (Dunlop & Fratzl, 2010 ▸). Advances in structural techniques across many fields have led to several breakthroughs recently, even if many open questions remain. In the present paper, we provide an overview of selected recent developments with the aim of identifying possible directions of technical developments where the crystallographic community can make a fundamental difference to our understanding of these complex materials. In the remainder of the introduction, we introduce two different example biological materials: bone and shells. We then cover selected approaches used to study their structure in 3D. We have purposely been selective rather than comprehensive with specific emphasis on the possibilities offered by the immense improvement in X-ray light sources with the advent of fourth-generation synchrotrons and the challenges involving hierarchical biocomposites.

### 1.1. Bone

**Figure 1:**
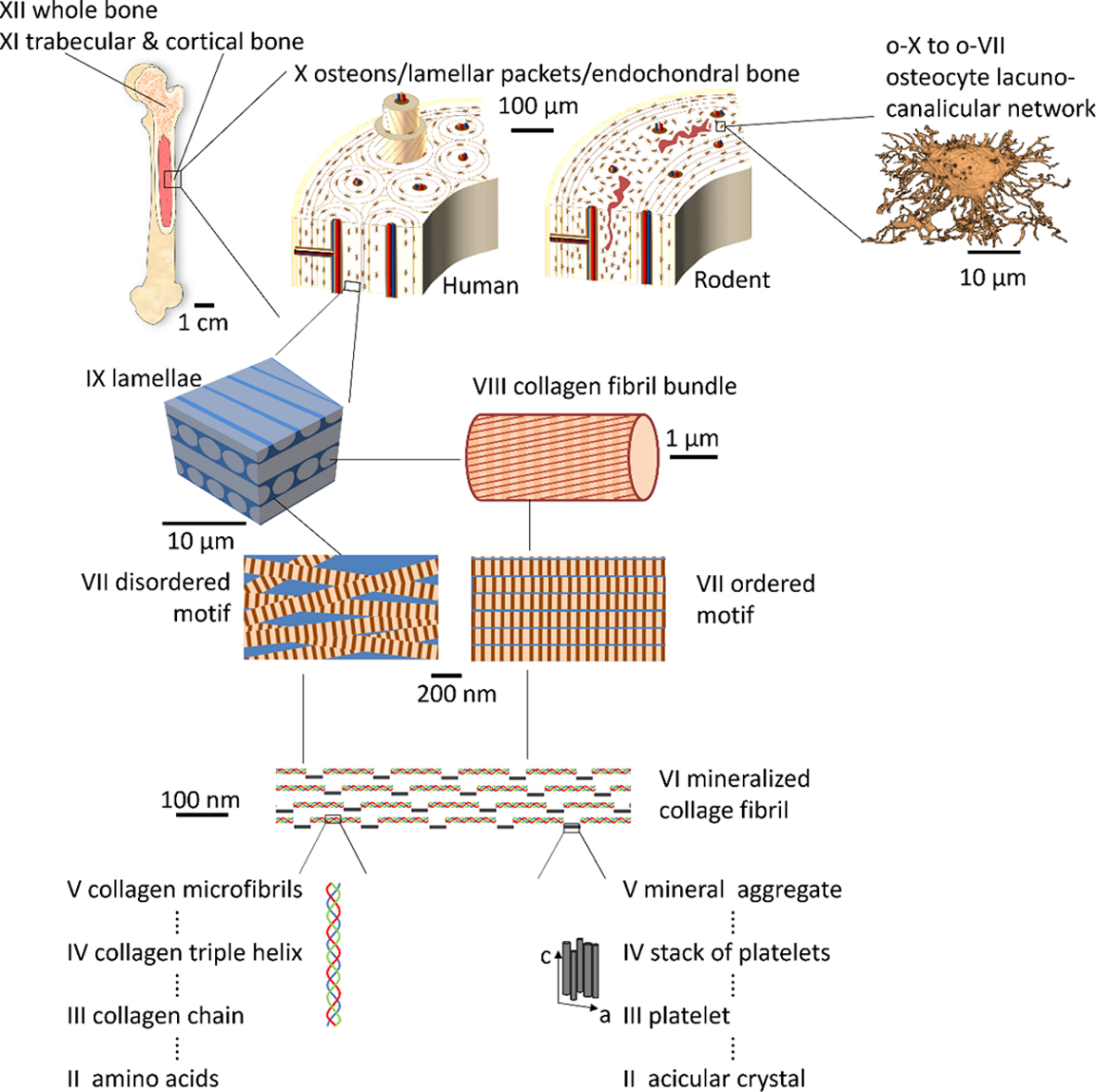
The hierarchical structure of bone reaches from the atomic (level I) to the macroscopic (level XII) length scale. See the text for a discussion of the structural elements.

Bone is an archetypical hierarchical material (Rodriguez–Palomo *et al.*, 2023 ▸; Wittig *et al.*, 2022 ▸; Wittig & Birkedal, 2022 ▸; Liu *et al.*, 2020 ▸; Eder *et al.*, 2018 ▸; Liu *et al.*, 2017 ▸; Wegst *et al.*, 2015 ▸; Stock, 2015 ▸; Wang & Gupta, 2011 ▸; Dunlop & Fratzl, 2010 ▸; Weiner & Wagner, 1998 ▸; McKee *et al.*, 2022 ▸; Buss *et al.*, 2022 ▸; Reznikov *et al.*, 2014 ▸, 2016 ▸) and has been studied for centuries (Havers, 1729 ▸). Technological developments in structural techniques have steadily refined our understanding of bone even if many open questions remain. Since several reviews have been published recently, here we will only outline the main features of bone structure, but we wish to underline that many variations occur. Bone has long been described as being hierarchical (Weiner & Wagner, 1998 ▸) with the number of identified hierarchical levels steadily rising (Reznikov *et al.*, 2018 ▸, 2014 ▸; Buss *et al.*, 2022 ▸). Here we use the hierarchy proposed by Reznikov *et al.* (2018 ▸). At the first levels, from the atomic (level I) to level V, we find the major building blocks of bone: collagen type I, water and the biomineral phase, which is nanocrystalline and apatite-like. Apatite is hexagonal, *P*6_3_/*m*, with *a* ≃ 9.4 and *c* ≃ 6.9 Å. The crystallites are anisotropic in size, displaying much sharper (00*l*) than (*hk*0) diffraction peaks. The mineral ‘particles’ have been described as plates (Weiner & Wagner, 1998 ▸) which is seemingly at odds with the hexagonal symmetry of the ‘crystallites’. Reznikov *et al.* (2018 ▸) used transmission electron microscopy (TEM) tomography to propose a model of bone mineral at the nanoscale that addresses this issue: they concluded that mineral platelet particles are formed as fractal aggregates of acicular crystals. This could conceivably occur by oriented attachment (De Yoreo *et al.*, 2015 ▸) or similar crystal–crystal interactions. The crystals aggregate further to mineral aggregates (level V) up to a few hundred nanometres in length (Reznikov *et al.*, 2018 ▸). On the collagen side, amino acids form collagen molecules (level III) that spontaneously self-assemble into triple-helices (level IV) that form microfibrils (level V) with a characteristic staggered packing of collagen triple-helices to result in fibrils with a stacking period (*D*-period) of ∼64–67 nm (Perumal *et al.*, 2008 ▸; Orgel *et al.*, 2006 ▸). Note that many additional biomolecules are present in bone, albeit in smaller quantities than collagen. At level VI, collagen and mineral meet to form mineralized collagen fibrils with the mineral *c* axis being predominantly co-aligned with the collagen long axis even if localized variations from this dominant theme may occur (Grünewald *et al.*, 2020 ▸). The mineralized collagen fibrils organize into motifs at the micrometre-scale with varying degrees of order (level VII). The ordered motif forms collagen fibril bundles (level VIII) that, together with the disordered motif, assemble into lamellae (level IX) at the ∼10 µm scale (Reznikov *et al.*, 2014 ▸, 2013 ▸). Also note that other assemblies are possible depending on the exact tissue type and biological context (Weiner & Wagner, 1998 ▸). The lamellar motif is the dominant one in the dense outer shell of long bones, called cortical bone (level X). Some animals, for example humans, undergo haversian remodelling that results in cylindrical motifs predominantly aligned along the bone axis. These motifs, osteons, were first described three centuries ago (Havers, 1729 ▸), but it remains an open question why some animals form them while others do not. Rodents, for example, do not undergo haversian remodelling and therefore have a different structure of cortical bone at the ∼100 µm scale (Fig. 1 ▸). Indeed, they present a central band originating from the initial bone formation process, called endochondral bone formation, containing remnants of mineralized calcified cartilage (Bach-Gansmo *et al.*, 2015 ▸; Bach-Gansmo *et al.*, 2013 ▸). The whole bone (level XII) also contains trabecular bone that acts as struts to add mechanical support. Unlike shells (next section), bone contains cells – osteocytes – housed in lacunae within the mineralized matrix. The lacunae are interconnected by canaliculi, just a few hundred nanometres in diameter, to form a vast network of cells that orchestrate bone remodelling and interact with other organs; see, for example, the recent review by Robling & Bonewald (2020 ▸) and Section 2.

The anisotropic 3D arrangement of hierarchical elements across length scales results in highly anisotropic mechanical properties of bone (Seto *et al.*, 2008 ▸; Koester *et al.*, 2008 ▸; Stockhausen *et al.*, 2021 ▸). At the materials level, bone has dissipative, self-healing properties (Fantner *et al.*, 2005 ▸). The self-healing properties are enabled by non-collagenous biomacromolecules through interactions with calcium ions (Wang & Gupta, 2011 ▸; Gupta *et al.*, 2007 ▸; Fantner *et al.*, 2005 ▸). The effective moduli of the mineral and collagen have been studied by high-energy small-angle X-ray scattering (SAXS) and X-ray diffraction (XRD) under load (Almer & Stock, 2007 ▸, 2005 ▸). These revealed that the mineral phase in the biocomposite is under compressive residual stress. Furthermore, applied mechanical deformation results in deformations of the mineral as well as the collagen fibril (Gupta *et al.*, 2006 ▸). Indeed, the mineralization process itself changes the collagen *D*-spacing at a level that would correspond to applied forces in the megapascal range (Ping *et al.*, 2022 ▸). The intimate connection between collagen and mineral in combination with the hierarchical structure thus defines bone mechanical properties. Much remains to be learned, however, of how individual components and hierarchical elements impact bone mechanics, which in our view is a prerequisite in the development of quantitative models for the prediction of bone mechanical properties including fracture risk.

It has recently been emphasized that, although bone has an archetypical structure, important spatial variations occur, for example, at cement lines bordering osteons, in the perilacunar matrix and many others (Rodriguez–Palomo *et al.*, 2023 ▸; Wittig & Birkedal, 2022 ▸). This calls for a focus on ensuring that any study of ‘bone’ is placed within its biological context. In turn, this indicates that future research will benefit from spatially resolved studies bridging length scales, which is our motivation for highlighting such techniques in the present contribution.

### 1.2. Shell

Another example of a hierarchically structured biomaterial are shells. They feature an astonishing diversity in terms of macrostructural motifs, first reported nearly 100 years ago (Bøggild, 1930 ▸) and still the subject of intensive research. The most commonly investigated structures are the prismatic structure (Olson, Metzler *et al.*, 2013 ▸; Duboisset *et al.*, 2022 ▸; Dicko *et al.*, 2022 ▸; Dauphin *et al.*, 2019 ▸; Nudelman *et al.*, 2007 ▸) as well as the nacreous layer (Metzler *et al.*, 2007 ▸; Nudelman, 2015 ▸; Addadi *et al.*, 2006 ▸; DeVol *et al.*, 2015 ▸). However, other structures such as the cross-laminar structure or the chalky layer structure are also present (Checa, 2018 ▸). Though calcite seems to be the predominant polymorph for the prismatic layer and aragonite for nacre, cross-laminar and chalky layer structure, the polymorph selectivity has been shown to be flexible, at least in the case of pearl nacre (Cuif *et al.*, 2022 ▸). The formation of the shell involves a nanogranular, amorphous calcium carbonate (ACC) precursor (Weiss *et al.*, 2002 ▸; Nassif *et al.*, 2005 ▸; DeVol *et al.*, 2015 ▸; Duboisset *et al.*, 2022 ▸; Dicko *et al.*, 2022 ▸; Grünewald *et al.*, 2022 ▸; Addadi *et al.*, 2003 ▸; Dauphin, 2008 ▸), which appears to be stabilized by organic molecules prior to crystallization. A further complicating factor is that the whole formation process of the shell takes place in sea water with the associated constraints on *T*, pH and availability of ions. While the mineral forming is protected by a thin membrane [the Marsh membrane (Marsh & Sass, 1983 ▸; Cuif & Dauphin, 2018 ▸)] which creates an enclosed space filled with extrapallial fluid between the prism and the Marsh membrane, the mineralization process is subject to constant dissolution and precipitation effects due to the local environment of this extrapallial fluid.

**Figure 2:**
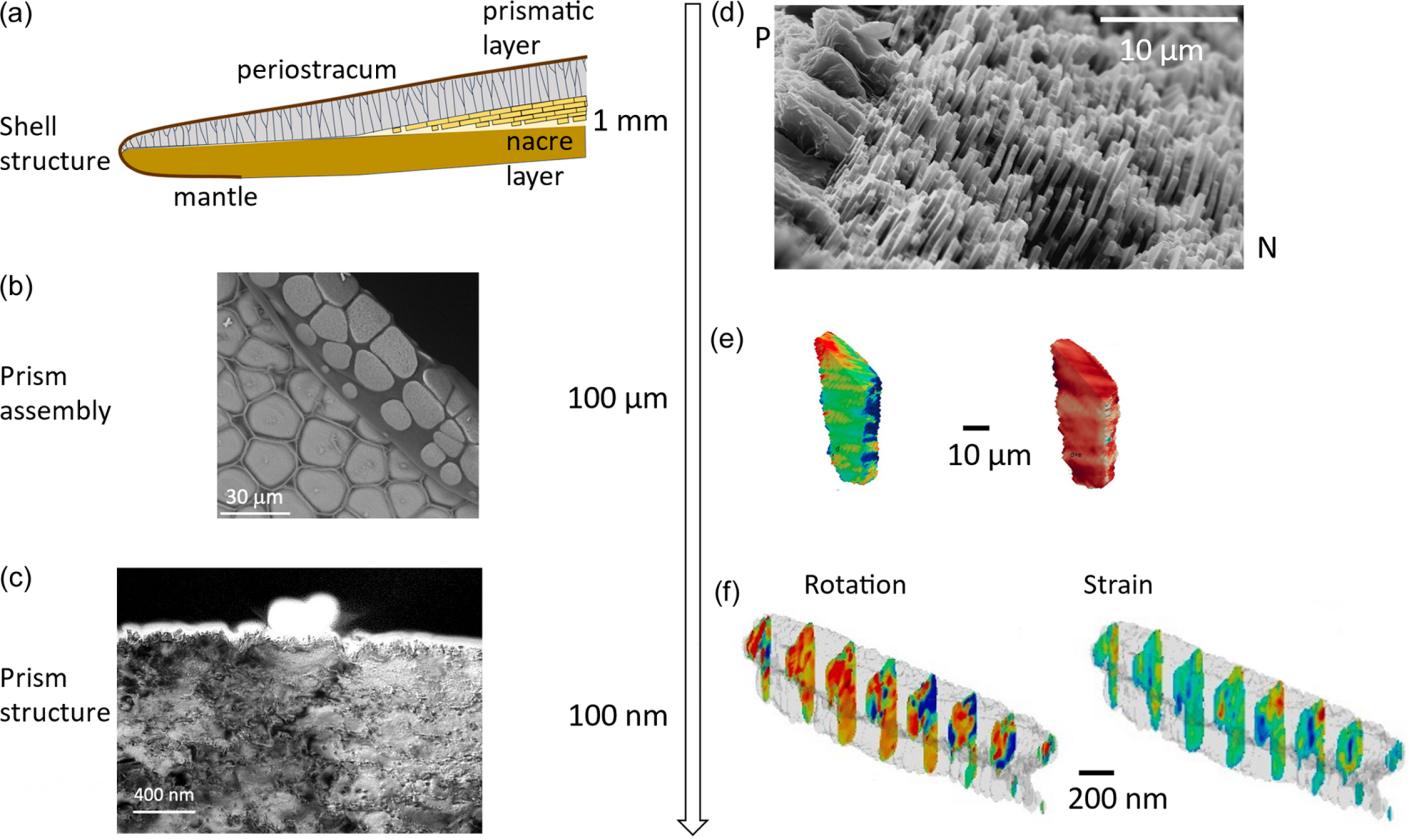
Structure of the prismato-nacreous mollusk shell. (*a*) Schematic overview of the shell structure. The shell is comprised of a calcareous prismatic and nacreous layer, sandwiched between the organic mantle and periostracum layer. (*b*) Zoomed-in image of the prismatic layer shows the early stage mineralizing units (discs) sitting on the periostractic membrane and their subsequent, space-filling assembly in the later stages. (*c*) Further zoom shows a TEM cross-section, showing the layered growth motif under a presumed nucleation centre (bright nodule). (*d*) Prism–nacre interface in abalone shell by SEM (Birkedal, private communication). (*e*) Dark-field X-ray microscopy allows the study of lattice rotation and strain in full prismatic assemblies. (*f*) Bragg ptychography enables the visualization of lattice rotations and strain with very high, sub-100 nm spatial resolution across the full cross-section of an early stage disc. Panels (*b*) and (*c*) is reproduced from Duboisset *et al.* (2022 ▸) (https://creativecommons.org/licenses/by-nc-nd/4.0/), panel (*e*) is reproduced from Schoeppler *et al.* (2022 ▸) (https://creativecommons.org/licenses/by-nc/4.0/), and panel (*f*) is reproduced from Mastropietro *et al.* [(2017 ▸). *Nat. Mater.***16**, 946–952. Springer Nature].

As in bone, the key to understand the structure–function relationship in shell is to understand their organization at different length scales, indicated in Fig. 2 ▸. These length scales are common across the many different shell microstructures and the challenges are thus general and not restricted to the prismato-nacreous mollusk-shell example of Fig. 2 ▸. Key questions that remain open in this context include the sequence of formation, the impact of local environmental conditions (pH, temperature, local ion concentrations, biomolecules secreted by the animal) on the process. Although this review will not address the scientific challenges in detail, we want to highlight the fact that the shell contains a complex, single-crystal like mineral, produced from an amorphous precursor and further organized into different hierarchical structural elements (*e.g.* nacre that consists of plates arranged in 3D, Fig. 2 ▸), which also exhibit a variety of calcium carbonate polymorphs. One particular challenge in this context is to study the transformation from amorphous to crystalline polymorphs. XRD is an excellent tool to study amorphous compounds in shell (Grünewald *et al.*, 2022 ▸); however, the sensitivity and spatial resolution are somewhat limited compared with the study of crystalline signals, requiring complementary methods like electron microscopy (de Frutos *et al.*, 2023 ▸) or ptychographic tomography (Section 2) to exploit electron density differences, or X-ray absorption techniques to study changes in the atomic structure.

In the following, we will examine how different 3D methods allow us to access different hierarchical features in selected biological systems. For shell, for example, full-field tomography (see Section 2) methods enable us to study the growth kinetics and evidence a thermodynamically driven grain growth (Bayerlein *et al.*, 2014 ▸). Further high-resolution holotomography (see Section 2) enables the study of the impact of defects on the formation of the nacreous layer (Beliaev *et al.*, 2021 ▸) and, in general, enables a first attempt to model the growth behaviour from thermodynamic principles (Schoeppler *et al.*, 2018 ▸; Zlotnikov & Schoeppler, 2017 ▸). High-resolution powder diffraction on bulk samples has shown widespread lattice distortions due to biomolecules occluded within the seemingly single crystals (Pokroy *et al.*, 2007 ▸, 2006 ▸, 2004 ▸). Focusing on the spatial distribution of the crystalline arrangement of submicrometric domains, coherent XRD methods enable, for the first time, observation of the presence of multiple crystalline domains in the prismatic layer forming (Mastropietro *et al.*, 2017 ▸); further, larger scale investigations with dark-field X-ray microscopy show the lattice strain distribution over larger, functional units (Schoeppler *et al.*, 2022 ▸).

## 2. From macrostructure to nanostructure: tomography

The 3D structure of materials can by studied by computed tomography (CT) (Withers *et al.*, 2021 ▸; Stock, 2008 ▸). These techniques are immensely powerful and currently undergoing a revolution within in house instrumentation, synchrotron facilities and neutron experiments, enabling higher resolution, larger specimens, better contrast, drastically improved signal to noise and/or faster measurements. Since biominerals have hierarchical structures (Section 1), tomography provides an ideal window into their structure and – with developments in throughput and resolution, discussed in the following two subsections – provides outstanding opportunities for multiscale imaging of biominerals (Wittig *et al.*, 2022 ▸). Neutron imaging shows promise, especially through correlative investigations with X-rays (Østergaard *et al.*, 2023*b* ▸; Törnquist *et al.*, 2022 ▸, 2021 ▸; Guillaume *et al.*, 2021 ▸). With the increased coherence of fourth-generation synchrotron sources, phase contrast imaging increases in use and allows very rapid imaging (García–Moreno *et al.*, 2021 ▸) also of living animals (Mokso *et al.*, 2015 ▸; Walker *et al.*, 2014 ▸). Neutron propagation based phase contrast has recently been demonstrated on biological materials, including bone (Østergaard *et al.*, 2023*a* ▸; Paganin *et al.*, 2023 ▸).

### 2.1. High-throughput tomography: statistics and *in situ* mechanics

Biological materials are intrinsically variable. Therefore, it is necessary to ensure that conclusions on the structure and/or effects of interventions, diseases or genetics are supported by studying a sufficiently large number of specimens. For standard laboratory µCT, with voxel sizes down to ∼6 µm, this is easily achievable (*e.g.* Wittig *et al.*, 2016 ▸; Bach-Gansmo, Wittig *et al.*, 2016 ▸; Bach-Gansmo, Brüel *et al.*, 2016 ▸), but for higher-resolution studies (*e.g.* of osteocyte lacunae), synchrotron tomography strongly increases throughput with the added benefit that the near-monochromatic nature of the synchrotron beams used in such experiments resolves issues such as beam-hardening (Withers *et al.*, 2021 ▸; Stock, 2008 ▸). Given the high flux of synchrotron light sources, hundreds of specimens can be investigated (Hoac *et al.*, 2020 ▸; Wittig *et al.*, 2016 ▸; Bach-Gansmo, Wittig *et al.*, 2016 ▸; Bach-Gansmo, Brüel *et al.*, 2016 ▸; Mader *et al.*, 2015 ▸). For bone, for example, this has resulted in deep insights into osteocyte lacunae, as recently reviewed (Rodriguez–Palomo *et al.*, 2023 ▸). Synchrotron µCT experiments enable a large, millimetre-sized field of view (FOV) while retaining good, micrometre spatial resolution. This was used to study the formation process of oyster shell. By exploiting the fact that the axis along the prism long axis serves as a temporal formation history, Bayerlein *et al.* (2014 ▸) could show that a thermodynamically driven, competing growth process occurs.

The excellent capabilities for 3D imaging with micrometre-scale resolution offers insights into bone mechanics, which are strongly impacted by bone hierarchical organization at these length scales. With careful control over dose (see also Section 6.1), synchrotron imaging has provided detailed insights into fracture propagation in bone and factors influencing bone toughness (Koester *et al.*, 2008 ▸; Launey *et al.*, 2010 ▸; Barth *et al.*, 2010 ▸; Zimmermann *et al.*, 2009 ▸, 2015 ▸).

#### 2.1.1. High-resolution tomography: holotomography and ptychography

To obtain higher resolution than the few-hundred nanometre voxel sizes of ‘classical’ synchrotron tomography, alternative measurement schemes must be employed. Two such techniques will be mentioned here: holotomography (Cloetens *et al.*, 1999 ▸) and ptychography (Dierolf *et al.*, 2010 ▸). These techniques are currently undergoing respective revolutions owing to the strongly increased coherence of fourth-generation synchrotron sources with predictions of orders-of-magnitude improvement in performance (Streun *et al.*, 2018 ▸).

Holotomography is based on the expansion of the X-ray beam from a pre-sample focus and then sampling the transition from contact – pure absorption – to the Fresnel – diffraction – regime (Cloetens *et al.*, 1999 ▸), allowing back-calculation of the X-ray refractive index and hence the sample electron density. This provides excellent information across a broad spectrum of materials including, for example, neuroimaging (Kuan *et al.*, 2020 ▸; Andersson *et al.*, 2020 ▸; Khimchenko *et al.*, 2018 ▸) nacre (Beliaev *et al.*, 2021 ▸, 2020 ▸) and bone (Hesse *et al.*, 2015 ▸; Varga *et al.*, 2013 ▸; Pacureanu *et al.*, 2012 ▸; Langer *et al.*, 2012 ▸; Wittig, Laugesen *et al.*, 2019 ▸).

Ptychography is a lens-less imaging technique, initially conceived for electron microscopy (Hoppe, 1969 ▸), later ‘rediscovered’ for X-ray imaging (Thibault *et al.*, 2008 ▸; Rodenburg, 2008 ▸) and extended towards a now rather mature nanotomography technique where the image is built up of overlapping coherent scattering patterns from sample to build 2D projection images of the sample that can then be tomographically reconstructed (Dierolf *et al.*, 2010 ▸). An excellent overview of the development of this technique is given by Guizar-Sicairos & Thibault (2021 ▸). Ptychographic tomography provides very high spatial resolution (Holler *et al.*, 2019 ▸; Holler *et al.*, 2014 ▸; Holler, Guizar-Sicairos *et al.*, 2017 ▸) but is somewhat limited in sample size even if lamino­graphic implementations allow larger in-plane samples sizes (Holler *et al.*, 2019 ▸). Implemented in TEM, it even affords sub-atomic resolution (Nguyen *et al.*, 2024 ▸). It can be combined with X-ray fluorescence CT (XRF-CT; next section) and with cryo-preservation affords exquisite insights into the interior of cells (Deng *et al.*, 2018 ▸, 2015 ▸).

Both ptychography and holotomography yield a measure of the average electron density in each voxel, which is highly useful for the study of biological materials since this information is well nigh impossible to obtain in 3D and at this spatial resolution otherwise (Hesse *et al.*, 2015 ▸; Birkbak *et al.*, 2016 ▸). As the electron density is directly related to the reconstructed phase information in the sample [see, for example, Diaz *et al.* (2012 ▸) for a further description], the distinction between different polymorphs could be made via their electron density difference. The accurate measurement of the spatial variation in electron density for example allowed estimation of the protein content within the central part of sponge glass spicules (Birkbak *et al.*, 2016 ▸).

For bone, the increased resolution and quantitative recovery of the electron density has in particular afforded insights in the osteocyte lacuno-canalicular network and the associated bone matrix (Hesse *et al.*, 2015 ▸; Varga *et al.*, 2013 ▸; Pacureanu *et al.*, 2012 ▸; Langer *et al.*, 2012 ▸; Wittig, Laugesen *et al.*, 2019 ▸; Ciani *et al.*, 2018 ▸) with similar results for dentine (Zanette *et al.*, 2015 ▸). This showed that the mineral matrix immediately adjacent to the osteocyte canaliculi has a larger electron density – interpreted as a higher degree of mineralization – than the matrix further away (Hesse *et al.*, 2015 ▸), reflecting the emerging realization that bone is heterogeneously mineralized (Rodriguez–Palomo *et al.*, 2023 ▸; Wittig & Birkedal, 2022 ▸). It also enabled the discovery that the canalicular network contains junctions where several canaliculi meet (Wittig, Laugesen *et al.*, 2019 ▸), showing that the cellular network in bone is much more complex than previously believed.

The combination of high resolving power, quantitative electron density sensitivity and a relatively large FOV has also been appealing for the study of shells. This has enabled insights into how morphological ordering drives the formation of nacre (Beliaev *et al.*, 2021 ▸, 2020 ▸). One example where the electron density sensitivity has been exploited is the study by Ihli *et al.* (2021 ▸). They show how hydration of organic layers is used to dynamically change the structure of brachiopod shell (note that brachiopods are not mollusks but also have hierarchically structured shells). Potentially, this approach can also be used to study the distribution of amorphous and crystalline compounds owing to their difference in electron density.

## 3. Multimodal imaging: XRD-CT and XRF-CT

The ‘direct’ imaging techniques described in the preceding section are very powerful but do not directly provide information on molecular- to atomic-level structures nor on spatial variations in chemical composition. Scattering and diffraction provide information on the local molecular- to atomic-level structure whereas XRF affords quantitative insights into chemical composition. These techniques have been used to obtain spatially resolved information in 2D by raster scanning a thin sample through a pencil X-ray beam. With improving light sources, optics and detectors, the resolution and power of such methods have steadily improved. For example, SAXS – pioneered by Fratzl and coworkers (Rinnerthaler *et al.*, 1999 ▸; Fratzl *et al.*, 1991 ▸) – provides information on the hierarchical structure of bone averaged over a volume defined by the beam size and the thickness of the sample. The SAXS curve provides information related to the nanocrystal thickness and the collagen *D*-period as well as providing valuable information on the orientation of the nanoscale building blocks (see Section 4). Scanning XRF provides information on oligo elements, *e.g.* for bone, elements such as Zn and Sr are of particular interest because of their involvement in bone biomineralization (Christensen *et al.*, 2022 ▸; Wittig, Palle *et al.*, 2019 ▸; Rasmussen *et al.*, 2020 ▸; Pemmer *et al.*, 2013 ▸; Bleuet *et al.*, 2008 ▸; Dejea *et al.*, 2023 ▸; Silva Barreto *et al.*, 2020 ▸).

These techniques can – under the assumption of signal additivity – be extended to 3D tomography by combining scanning and rotation of the specimen. This resulted in XRD-CT that, with monochromatic X-rays, was first implemented at synchrotrons to study bone (Stock *et al.*, 2008 ▸) quickly followed by other applications (Bleuet *et al.*, 2008 ▸) involving multiphase biominerals (Leemreize *et al.*, 2013 ▸), see also the early review by Birkbak *et al.* (2015 ▸). The basic assumption is that each voxel contains a powder, *i.e.* that the crystallite size is significantly smaller than the beam size. This is essentially always the case for bone, which has very small crystallites. Combined with Rietveld refinement of reconstructed diffraction patterns (Frølich & Birkedal, 2015 ▸), XRD-CT affords detailed information, for example, on crystallite sizes by Scherrer peak-broadening analysis (Frølich *et al.*, 2016 ▸). It is straightforward to extend to SAXS-CT (Schroer *et al.*, 2006 ▸). With modern large detectors, it is even possible to collect ‘good enough’ SAXS and XRD information concurrently on the same detector to allow detailed insights of both the nano (from SAXS) and the atomic (from XRD) scale (Grünewald *et al.*, 2023 ▸), which drastically improves throughput and allows for accurate voxel based correlation of crystalline and nanostructural features. XRD-CT is particularly appealing due to its potential for *in situ* experimentation, especially when implemented with high-energy X-rays (Beale *et al.*, 2014 ▸). XRF-CT (de Jonge & Vogt, 2010 ▸; de Jonge *et al.*, 2010 ▸) provides 3D maps of the element composition throughout the specimen. With nano-focused X-ray beams, very high spatial resolution approaching the 100–200 nm scale can be achieved (Palle *et al.*, 2020 ▸). In practice, the sample diameter is limited by self-absorption of the emitted X-rays for biomineral samples.

A number of excellent works have used these techniques to shed light on biological materials [see, for example, the recent review on bone and similar materials (Rodriguez–Palomo *et al.*, 2023 ▸)]. Here we will only highlight two recent works. Wittig, Palle *et al.* (2019 ▸) combined XRF-CT and XRD-CT to study human osteonal bone. They established that the bone nanocrystals close to the osteon canal had different peak broadening – interpreted as smaller crystallite sizes – than in the ‘normal’ bone further away from the canal. They speculated that this may be a result of variations in the expression of mineralization-controlling biomolecules. Two of the present authors recently confirmed these results and showed that the border of the osteon, called the cement line, has different structural organization and biomineral properties than the surrounding bone (Grünewald *et al.*, 2023 ▸). These results demonstrate that bone is spatially heterogeneous. This emphasizes the need for spatially resolved experiments and we expect that future work will establish the origins of these heterogeneities as well as their impact on, for example, bone mechanics.

## 4. Orientation in 3D: tensor tomography

Scattering techniques are very well suited to study the orientation of anisotropic building blocks, smaller than the real-space resolution. Probing atomic distances and the orientation of crystalline phases is done with XRD, also called wide-angle X-ray scattering (WAXS). Nanostructures can be probed with SAXS, the momentum transfer (*q*) resolved scattering information allows us to probe specific length scales. Dark-field X-ray imaging is a full-field technique, which is sensitive to scattering from sub-pixel sample microstructures collected in the integrated USAXS/SAXS signal. If the azimuthal variation of all those scattering signals can be probed, then the orientation direction and degree of orientation of the underlying structure can also be probed. This is advantageous because it is not necessary to increase the resolution in tomography as described in the previous section for XRD-CT.

One scattering measurement with a 2D detector provides a 2D section through 3D reciprocal space. With respect to orientation information, it only provides information about the 2D projection of the underlying 3D orientation. The 3D orientation can be obtained from 2D sections of a sample by measuring scattering patterns at different rotations and reconstructing the out-of-plane orientation (Yifei *et al.*, 2010 ▸; Seidel *et al.*, 2012 ▸; Georgiadis *et al.*, 2015 ▸). As outlined in Section 3, scanning X-ray techniques can be combined with CT to resolve the inside of 3D samples. However, this standard approach is applicable only if the scattering is invariant with respect to the tomographic rotation, which is the case for isotropic scatterers or samples with structural symmetry around the rotation axis. Under strict assumptions on the sample (*i.e.* known shape and dimensions of the scatterers as well as only slow variations of orientation confined in one plane) the orientation distribution can be obtained from a single rotation axis (Skjønsfjell *et al.*, 2016 ▸). To reconstruct the orientation information, which is contained in a scattering experiment of anisotropic structures, in each voxel, a tensor rather than a scalar value is reconstructed. Hence these techniques are referred to as tensor tomography. For a full sampling of the 3D reciprocal-space map, measurements around two rotation axes need to be acquired. A complete sampling is usually restricted by the geometry of the setup, resulting typically in a missing wedge in the second rotation axis at tilt-angles larger than ∼45°. Different reconstruction algorithms have been reported for directional dark-field tensor tomography (sometimes also called X-ray scattering tensor tomography) (Wieczorek *et al.*, 2016 ▸; Kim *et al.*, 2020 ▸; Malecki *et al.*, 2014 ▸), for SAXS tensor tomography (Schaff *et al.*, 2015 ▸; Liebi *et al.*, 2015 ▸; Gao *et al.*, 2019 ▸; Nielsen *et al.*, 2023 ▸) and were extended to include WAXS/XRD tensor tomography based on a single diffraction peak (Grünewald *et al.*, 2023 ▸, 2020 ▸). The field continues to develop, for example, through very recent reports of multi-Bragg-peak based orientation analysis, with the aim of a fully quantitative description of more complex, full orientation distribution functions in an approached named texture tomography (Frewein *et al.*, 2024 ▸).

In particular SAXS tensor tomography has been used to study the hierarchical structure of bone, extracting the orientation of mineralized collagen in human trabecular bone (Liebi *et al.*, 2015 ▸), the re-orientation of mineralized collagen during bone healing around degrading Mg implants (Liebi *et al.*, 2021 ▸) and the orientation of bone near cement lines (Grünewald *et al.*, 2023 ▸). Directional dark-field tensor tomography provides information on larger structures, such as microtubuli in shark teeth (Kim *et al.*, 2020 ▸). A combination of XRD tensor tomography with XRD-CT has been used to study the orientation of hy­droxy­apatite crystallites near the ossification front in piglet femoral condyle (Mürer *et al.*, 2021 ▸), while the combination of SAXS and XRD tensor tomography revealed a localized difference in orientation distribution in human lamellar bone from the nanoscale scattering of mineralized collagen and the biomineral crystal structure (Grünewald *et al.*, 2020 ▸). See the work by Rodriguez–Palomo *et al.* (2023 ▸) for a summary of applications of tensor tomography involving bone.

## 5. Crystalline properties with high angular and spatial resolution – Bragg coherent diffraction techniques

The crystalline properties of materials and their relative anisotropy for different crystalline orientations add an important element to the outstanding tunability of hierarchically structured materials. A crystal in this context is defined as a periodic arrangement of atoms, ions or molecules, making up a distinct unit (unit cell), whose repetition yields a larger, coherent 3D crystal. In the real world, these perfect, large crystals are often disturbed by defects, impurities and strain, all of which are important characteristics and help in understanding the genesis of the crystalline matter.

To obtain information on the crystalline properties with XRD, a set of crystalline planes and their respective reciprocal lattice vector need to match the Bragg vector of the incident wave in order to produce a reflection. The important difference to the methods presented in Sections 3 and 4 is that Bragg coherent diffraction imaging (Bragg CDI) techniques aim to investigate a single, isolated Bragg peak, thus limiting its application to rather well crystallized materials, excluding, for example, the study of bone mineral at the current state of development. By slightly rotating and tilting the lattice vector, different parts of the reciprocal space can be reconstructed and a full characterization of the crystalline properties of this particular set of lattice planes can be carried out because the diffraction contrast is associated with the density of the investigated crystal and the projection of its displacement field on the Bragg vector. This can be exploited to produce a 2D image of the crystalline properties by scanning a finely focused beam across the sample. This technique has been used traditionally to study the crystalline properties of biomaterials such as shells (Olson, Blonsky *et al.*, 2013 ▸; Duboisset *et al.*, 2022 ▸; Metzger *et al.*, 2014 ▸), sea urchin (Killian *et al.*, 2009 ▸) or the brittlestar lens (Polishchuk *et al.*, 2017 ▸), but can, in principle, also be used to study amorphous compounds within a crystalline matrix in shells (Grünewald *et al.*, 2022 ▸), although great care is necessary during the data treatment of such a complex diffraction signal.

The principle of studying individual Bragg peaks is combined with an X-ray lens in 3D X-ray dark-field microscopy to produce an image of the crystalline reflection and use a tomographic approach to produce a 3D image of crystalline properties (Simons *et al.*, 2015 ▸). Here, the attainable spatial resolution is limited by the beam size and can reach ∼100 nm. A particularly attractive aspect of this technique is the possibility to isolate a single crystalline reflection and, in this way, ‘zoom’ into a sample without the need to prepare a small sample. A recent example is the study of lattice defects in the prismatic layer of bivalves (Schoeppler *et al.*, 2022 ▸).

One very important limitation of all the aforementioned scanning approaches is a spatial resolution that is limited by the available beam size. Although small hard X-ray beams of sub-50 nm are routinely available at modern synchrotron facilities, covering a large FOV in 2D (let alone 3D) becomes prohibitive for hierarchical imaging in terms of both scan time as well as deposited dose on the sample. One way to overcome this limitation is to use the lens-less CDI approaches presented in Section 2 where the attainable resolution is linked not to the beam size but to the sampling of the signal in reciprocal space. These approaches exploit the fact that the phase, which combines in the Bragg diffraction case both strain and electron density sensitivity, is usually lost in a conventional XRD experiment and poses the so-called phase problem. However, by illuminating the sample with a fully coherent X-ray beam and imposing strategies to either impose constraints on the extent of the sample Bragg CDI or provide additional diversity by oversampling the sample with translations across the beam (Bragg ptychography). With this additional information, the phase information can be recovered via iterative phase retrieval algorithms. To obtain 3D information on the crystalline properties, the sample needs to be rotated. As all of the information on the 3D structure of the particular domain is contained in the Bragg reflection, sampling of this single reflection with sufficient oversampling is sufficient to obtain the full 3D information (Williams *et al.*, 2003 ▸). Two main approaches can be differentiated here: Bragg CDI, where a plane-wave illumination of an isolated object within the coherent volume of the X-ray beam is necessary; or Bragg ptychography, where the lateral FOV can be extended beyond the coherent volume of the X-ray beam by raster sampling with a step size smaller than the beam size and introducing oversampling to the reconstruction of the phase (Godard *et al.*, 2011 ▸). In special cases, 3D information can even be retrieved from a single angle with a back-projection reconstruction approach (Hruszkewycz *et al.*, 2017 ▸). Based on this approach and its mathematical reformulation of the reconstruction approach, further advancements have been to made to enable probe reconstruction (Li *et al.*, 2021 ▸) and position refinement (Li *et al.*, 2022 ▸).

All these techniques were pioneered in the early 2000s and have been applied mostly to engineering materials with relatively perfect crystals. This is mostly owed to the relatively low coherent flux of the synchrotron sources of the time. However, a real revolution has arrived in recent years with fourth-generation synchrotron sources. The first sources in full user operation [MAX IV in Sweden (Tavares *et al.*, 2018 ▸) and the ESRF-EBS in Grenoble (Raimondi *et al.*, 2023 ▸)] have demonstrated ∼50-fold improvement in brilliance and an improvement in coherent flux on the same order of magnitude. This has enabled us to significantly improve the signal quality and reduce the time required to collect a dataset. This has furthermore enabled us to alleviate some of the stability constraints connected with long experiments (Li *et al.*, 2022 ▸), but has also brought up radiation damage concerns. This has eventually prompted the development of techniques that try to be more dose-efficient by encoding extra information due to selective modulation of the incoming wavefront (Calvo-Almazan *et al.*, 2024 ▸).

In terms of hierarchical imaging, CDI techniques are used to probe crystalline properties on a very local scale (with a few tens of nanometres spatial resolution) in a volume of a few cubic micrometres. Their use is complementary to electron microscopy techniques where X-ray techniques, while being of lower spatial resolution, enable non-destructive 3D investigations, higher strain and orientational sensitivity, as well as the potential for *in situ*/*operando* studies. The 3D information accessible in a Bragg CDI/ptychography dataset is quantitative strain and crystalline orientation with high resolution and sensitivity [strain ∼10^−4^, orientation ∼10^−3^ degrees with ∼50 nm spatial resolution (Chamard *et al.*, 2015 ▸; Li *et al.*, 2021 ▸; Pateras *et al.*, 2015 ▸)]. We want to underline that these values are always sample- and experimental-setup-dependent and need careful evaluation for each sample system, in particular the spatial resolution. One particular interesting feature is the characterization of the coherence length of a crystalline domain from the phase information. This set of information allows us to tackle some of the important pending questions in the field of biomineralization: how do we reconcile some of the seemingly contradictory aspects of classical nucleation theory with nonclassical crystallization mechanisms from an amorphous precursor particle, like the crystalline domain size and the imprint of the amorphous particles? Which role do organic inclusions and foreign ions play in the shaping and growth of biominerals? How do biomineralizing organisms form a large, coherent crystal while relying on small, amorphous precursor particles?

Examples for the application of Bragg CDI techniques on biomineral samples are comparatively sparse. Notable work has been presented by the Meldrum group on the characterization of strain fields inside of synthetic, calcite crystals with BCDI, elucidating the role of organics in templating (Ihli *et al.*, 2016 ▸); and further, the effects of organic inclusions on the crystalline structure (Ihli *et al.*, 2019 ▸). Another example is the work of the Chamard group. Here, the prismatic layer of the bivalve *Pinctada margaritifera* has been investigated in great detail (Mastropietro *et al.*, 2017 ▸), highlighting the granular structure previously observed (Dauphin, 2008 ▸; Addadi *et al.*, 2003 ▸; Weiss *et al.*, 2002 ▸), the extension of crystalline coherence across multiple granules as well as the presence of multiple slightly (∼0.2°) misaligned crystalline domains in the ‘single’ crystalline prismatic crystal. By employing the Bragg ptychography approach, a macroscopic, unprepared piece of shell from a juvenile specimen with a thin and active growing edge can be utilized and, due to the inherent reconstruction robustness of the ptychography approach over BCDI approaches (Huang *et al.*, 2011 ▸), comparatively strong gradients in the strain field can be reconstructed. Still, the requirement of a thin sample which fits into the longitudinal coherence (approximately a few micrometres) needs to be met. Another example using Bragg ptychography is the work of Berenguer *et al.* (2014 ▸), where the collagen fibrillar organization has been studied in rat tail tendons. By employing the first-order collagen diffraction peak from the 67 nm *D*-period of tendon collagen, the authors obtained images exclusively showing collagen fibres within the extracted piece of tendon and were able to determine the local strain of the collagen fibrils that was found to be centred around 66.67–67.13 nm with a maximum variation of 0.2% in *D*-spacing over 10 µm along a fibril (Berenguer *et al.*, 2014 ▸).

Although Bragg CDI techniques offer unparalleled, sub-100 nm 3D spatial resolution for Bragg X-ray imaging, the actual determination of spatial resolution remains an open challenge. If sharp features are present, these can be used to give an estimate. More generally, one-size-fits-all approaches like the Fourier shell correlation tend to be overly optimistic and do not account for varying resolution across the sample. While the authors do not have a proposition for a better solution, common sense and caution are usually good starting points for a good, truthful resolution estimate.

## 6. Challenges to multi-modal characterization

Though it is clear that multi-modal characterization methods like those presented above provide deep and important insights, their implementation and use may be limited by various challenges. We have identified three major ones, while recognizing that there may be more: radiation damage, data volumes generated in the experiments and sample preparation; we now discuss each in turn.

### 6.1. Radiation damage

The key limit to the amount that can be theoretically extracted from a sample is the total X-ray dose that a sample can tolerate before it is destroyed. While this has traditionally been a major concern for fields such as protein crystallography or soft-matter studies (Burmeister, 2000 ▸), the advent of X-ray free-electron lasers has made most materials susceptible to radiation damage and has led to the development of a totally new class of single-particle sample-delivery methods to enable diffraction before destruction (Chapman *et al.*, 2014 ▸). The challenge is equally emerging for many communities due to the new fourth-generation synchrotrons and though the ever more brilliant beams unlock the potential for exciting, fast *in situ*/*operando* studies in 3D, radiation damage needs to be managed to obtain meaningful results (Bras *et al.*, 2022 ▸).

At this point it is important to define a few quantities to aid in the discussion later. The deposited dose, *d*, is proportional to 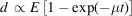 (Deymier-Black *et al.*, 2012 ▸), where *E* is the X-ray energy, *t* is the X-ray path length through the sample and μ is the linear attenuation coefficient (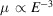). From this, we get 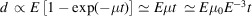, so that 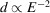. Thus, the deposited dose decreases with the energy squared, meaning that the choice of energy is a very important parameter when handling beam damage and even changes of a few kiloelectronvolts can make a big difference. This calls for a careful balance – different for each sample – of choice of energy in relation to available flux, signal to be measured and detector capabilities. Likewise, the radiation damage might also look different for each sample and requires careful assessment (Sauer *et al.*, 2022 ▸). For the case of bone, well established limits have been published, mostly taking the mechanical behaviour of the materials as an indicator for the onset of radiation damage (Barth *et al.*, 2010 ▸; Groetsch *et al.*, 2023 ▸). While the fading of a diffraction peak is traditionally associated with increasing disorder in a crystalline lattice, an increase/decrease in the SAXS can be attributed to alterations in certain components of a multi-phase system or the expulsion of water. More subtle traces of radiation damage can be the build up or release of strain, alterations of the particle size, and their distribution. It is thus imperative to understand the characteristics of the sample under study and the ‘fingerprint’ of radiation damage. So, while for some experiments (*e.g.* XRF-CT) an alternation of the nanostructure can be tolerated, the same damage is absolutely prohibitive for an experiment that focuses on this particular nanostructure with a SAXS-CT experiment. It is advisable to access the evolution of a scattering/diffraction signal with repetitive short exposures in the same sample position before deciding on the exposure time and number of total acceptable acquisitions. For methods such as SAXS/WAXS tensor tomography, which need the acquisition of many projections at rotations around two axes, it is advisable to monitor radiation damage by repeating a reference projection in the beginning, middle and end of the experiment.

The authors want to underline that there is no ready-made strategy to overcome the radiation damage issue but it is rather a combination of approaches that can help. For instance, there is strong dependency on dose rate and there is evidence that fast measurements with high dose rate are somewhat less invasive than the same dose accumulated over a longer time (de la Mora *et al.*, 2020 ▸). A small beam size with high flux provides very high flux density on small sample areas, sometimes one needs to strike a compromise between resolution and radiation damage (Silva Barreto *et al.*, 2023 ▸). For CDI techniques, introducing a structured illumination can, at the same time, reduce the flux density as well as increase the amount of information that can be extracted from a diffraction pattern (Odstrčil *et al.*, 2019 ▸; Calvo-Almazan *et al.*, 2024 ▸). Another strategy is to scan in an interlaced points pattern to mitigate the pre-damage to not-yet scanned points by the expanding cloud of free charge carriers. This could also inspire new ways of acquiring scanning tomographic data. Another avenue that is already widely exploited for protein crystallography or high-resolution nanotomography (Holler, Raabe *et al.*, 2017 ▸; da Silva *et al.*, 2017 ▸) is cryo-cooling of the sample. While not compatible with many *in situ* or *operando* schemes, it might provide the ability to measure hitherto impossible samples in 3D. Embedding of biological samples, as routinely done for allowing cutting of samples in histology, can also provide some additional stability to the sample, in particular for cases where radiation damage effects the macroscopic sample size, such as for shrinkage of the sample volume due to dehydration. Special resins which have a high resistance to radiation damage have recently been identified and where crucial for achieving high resolution in brain tissue (Bosch *et al.*, 2023 ▸).

We posit that radiation damage can also be mitigated by measuring data that are ‘good enough’ to address the question at hand. Here, especially imaging studies (be it in 2D or 3D) have the potential to benefit much more from spatial correlations/averages originating from either the measurement scheme or the sample. This in turn also requires beamlines that are able to acquire data fast enough and reduce unnecessary movement overheads. To fully harness the potential of such ‘sparse measurement strategies’ will require developments in data analysis approaches, possibly with the introduction of physical knowledge into, for example, reconstructions. In turn, this calls for careful reanalysis of the counting statistics and its propagation (*e.g.* through tomographic reconstruction). We believe that this area will provide a fruitful ground for future dose-minimizing experimental approaches.

In summary, radiation damage is a significant roadblock for the wide-spread application of 3D imaging techniques for biological materials. A proper recognition of the problem needs to happen before ways to overcome it can be studied. Some avenues to follow might be the increase of X-ray photon energy to minimize primary damage, the application of cryo-preservation to slow-down the spread of secondary damage, the application of new, fast scanning schemes to reduce the effective dose as well as taking data that are ‘good enough’ for the scientific question to address together with enhanced data treatment processes, adopted to sparse or noisy data.

### 6.2. The data deluge

The new methods that are currently developed – in particular in conjunction with fourth-generation synchrotron sources – open up new possibilities in terms of temporal and spatial resolution, as well as increasing the FOV, however these methods are inherently data-hungry. As an example, a tensor tomography experiment with a state-of-the-art detector running at 500 Hz (Johnson *et al.*, 2014 ▸) can accumulate 10 TB of data per day, whereas in full-field tomography the data rates can be higher reaching ∼30 TB per day easily. While this poses already big challenges on the synchrotron side in terms of data transfer, the post-processing on the user side is another challenge and might turn out to be a critical factor for the successful analysis and publication of an experiment. It is not uncommon to spend significantly more time reconstructing data than acquiring data, with measurement times being minutes to hours (and occasionally days) and reconstruction times being hours to weeks (excluding the ensuing lengthy data analysis).

One way of going about this problem is to only store data that have been reduced by means of integration, azimuthal regrouping or even tomographic reconstruction. While this is a viable strategy for standard measurements of a pre-defined kind, it is a dangerous strategy for experiments where feedback to the original raw data is necessary to understand some aspects of further data processing. One way to find a middle ground here would be to take the already employed compression schemes to the next level and instead of compressing the full 2D detector frames, only record the photon-counting events with their location and a time stamp and switch from a frame based to an event based detection that has been put forth for neutron detection and imaging (Losko *et al.*, 2021 ▸). This would of course require rethinking of some of our data processing strategies, but so does ever increasing optimization of our data processing pipelines towards distributed computing or GPU processing.

Another way to work around the problem is to simply store less data. In practice that would mean to only create a 3D dataset where the right sampling site and sample quality are ensured while working with 2D slices where they can be oriented in such a way to investigate the scientific question at hand.

An associated challenge is to visualize these higher-dimensional datasets. While the direction and degree of main orientation can still be visualized with a 3D glyph plot (Liebi *et al.*, 2015 ▸), they already become quite challenging to evaluate in detail once printed on paper. Higher-order quantities like a full orientation distribution function (3D quantity) for every voxel of a 3D space adds even more complexity (Nielsen, Tänzer *et al.*, 2024 ▸). Here, two strategies can be envisaged, either a dimensional reduction [extracting a certain feature and projection data or presenting a 1D distance plot (Liebi *et al.*, 2021 ▸; Grünewald *et al.*, 2023 ▸)]. This however also goes along with a reduction in data content. Another strategy could be to use animations of 3D spaces and a more refined 3D symbology to encode not only orientations, but more complete orientation distribution functions and thus to enable more information. This is in turn needs a rethinking of how current figures are embedded in publications.

A final thought on the amount of data, also linked to radiation damage, is to also rethink our way of conducting experiments. At present, the most common way of collecting data is to ensure that we ‘see’ the signal we want to see already in each single projection/diffraction pattern. This inevitably leads to ‘overexposed’ samples and thus an excess dose on the sample. The alternative approach is to define statistically meaningful counting limits for the whole tomogram as the dose fractionation theorem (Hegerl & Hoppe, 1976 ▸; Hoppe & Hegerl, 1981 ▸) ensures that the integral dose distributed over the sample is conserved. A practical example for this is 3D Bragg ptychography, where it is rather the total deposited number of photons than the individual diffraction patterns that serves as a guide-stick during the data acquisition (Mastropietro *et al.*, 2017 ▸). In turn, data treatment approaches need to be developed in a more statistics-centric fashion, including more realistic noise models and physics pre-knowledge of our samples to ensure the reconstructions and, in this way, reduce the necessary amount of data and dose deposited on the sample for a successful reconstruction.

### 6.3. Making the right sample for the right experiment

Sample preparation is – in our opinion – an often overlooked, almost always under-reported, but essential part of all experiments on biological materials and – we would argue – on materials in general. To increase success of the advanced experiments outlined in the preceding sections, sample quality is key, but in many cases, there is a lack of approaches to ensure sample quality prior to the actual synchrotron experiment. It is in practice a significant challenge to ensure that the samples produced actually allow us to address the scientific question raised. This involves issues such as how to ensure that the region of interest for the scientific question is present in the sample whilst ensuring that the sample fulfils the size/shape requirements of the experiments at hand. It is especially challenging to design samples suitable for several techniques. For example, self-absorption favours small sample diameters for XRF-CT while XRD-CT benefits from thicker samples providing a better signal to noise in the diffraction signal of each projection (Wittig, Palle *et al.*, 2019 ▸). These problems are accentuated by the complex multiscale structure of biological materials. In the case of bone, for example, the preparation of small samples (Ø ≃ 10 µm) is challenged by the presence of osteocyte lacunae that can render a small sample mechanically unstable during preparation. One of us recently presented a sample preparation protocol focusing on the preparation of samples of bone where a specific feature, which could be an osteocyte lacuna, a cement line or something different, had to be retained within the sample volume (Wittig *et al.*, 2024 ▸). This was achieved by a multistep procedure, where the region of interest was first identified in a larger sample volume by in-house 3D X-ray microscopy. This ensured proper placement of the sample on the sample mount. The sample was then cut into a cylindrical shape of the desired size using a sequence of steps quality checked by in-house 3D X-ray microscopy using a lathe inspired by Holler *et al.* (2020 ▸). This approach increased the success rate of sample preparation severalfold. It is our impression that success in sample preparation (*i.e.* procuring the right kind of sample for the right kind of technique) is heavily reliant on user experience. We therefore see a need for publication of more sample preparation protocols to ensure that the immense development efforts of one laboratory do not get lost in a few lines in an experimental section but become known and spread across the community to the benefit of all. This will require a change of mindset in the community so that greater value is placed on such efforts. We see the IUCr as being excellently positioned to drive this evolution, which will drastically democratize and improve the outcome of the advanced multimodal imaging methods discussed herein.

## 7. Outlook

3D X-ray imaging and scattering approaches have been shown to provide us with significant new insights into the structure of hierarchical biomaterials over the last decades. While some techniques have traditionally been reserved for synchrotron X-ray sources, laboratory based X-ray equipment has come a long way, up to the point that it can replace synchrotron measurements in some cases. An important question to ask at the beginning of each experiment is ‘which tools are needed to answer my scientific questions?’ There is a ‘pyramid of pain’ in terms of access to an instrument (laboratory/synchrotron), sample preparation, experimental planning and the data analysis step: does my question require a 2D or a 3D technique?

It is usually advisable to start with the least complicated technique and resort to more complex approaches only when really necessary. One very common observation is that the preparation work of the experiment accounts for more than 80% of the total time invested in the experiment, so proper preparation pays off very quickly.

Another aspect of good experimental planning is to use as much information as possible during the experiment. This is particularly important for synchrotron experiments where a combination of techniques is often possible. Collecting simultaneous scattering, diffraction and fluorescence tomography data is often possible with very little overhead. Also, at the data analysis step, the combination of multimodal and all the information contained in a scattering pattern allows for a more thorough understanding of the data. With the emergence of more and more coherent X-ray sources, the exploitation of coherent diffraction information can become a game changer when it comes to extracting more information without increasing the dose that is deposited on the sample or can be used to provide a low-dose overview of a sample before sampling specific points with a more focused beam.

When it comes to publishing, we call for publishing as complete protocols as possible to aid others in their attempts to prepare, conduct and analyse the experiments. It is our belief that the whole community profits from learning and that having established protocols lowers the entry barrier for novice users as well as increasing confidence in data. A bright example in this matter is the cryo-electron microscopy community where carefully describing experimental methods, dose, sample prep *etc.* has been the norm for many years now. In the same vein, we call for an increased exchange of data analysis code as well as experimental and analysed data. Some steps in these directions have been taken, for example by the present authors (Wittig *et al.*, 2024 ▸; Jensen *et al.*, 2022 ▸; Frølich & Birkedal, 2015 ▸; Nielsen *et al.*, 2023 ▸; Frewein *et al.*, 2024 ▸; Nielsen, Carlsen *et al.*, 2024 ▸), but should be extended far beyond the current level to the benefit of the full community.

One important lesson from the recent series of upgrades of synchrotron facilities is that, in order to exploit these new sources to their fullest, the beamlines and also the way they are currently operated need some rethinking. How can we provide flexible, multi-modal detection capability while maintaining the beamline so it is easy enough for inexperienced users? How can we implement a more seamless transition between different (coherent) measurement modes? How can we provide an easier access model to beam time and also enable new users to harness the full power of these beamlines? How can the synchrotron facilities help users in managing the data deluge and the task of analysing them?

With this topical review the authors have tried to outline the current state-of-the-art imaging of hierarchical materials, point out some directions for future developments and critically address the current challenges that are faced, with a particular focus on synchrotron applications. The field has seen immense discoveries over the last decade and the new synchrotron sources will only fuel these dynamics to help in the quest to understand the hierarchical organization of materials.

## Data Availability

The authors confirm that the data supporting the findings of this study are available within the article and its supplementary materials.
